# A New Extension of Thinning-Based Integer-Valued Autoregressive Models for Count Data

**DOI:** 10.3390/e23010062

**Published:** 2020-12-31

**Authors:** Zhengwei Liu, Fukang Zhu

**Affiliations:** School of Mathematics, Jilin University, 2699 Qianjin Street, Changchun 130012, China; zhengweil18@mails.jlu.edu.cn

**Keywords:** extended binomial distribution, INAR, thinning operator, time series of counts

## Abstract

The thinning operators play an important role in the analysis of integer-valued autoregressive models, and the most widely used is the binomial thinning. Inspired by the theory about extended Pascal triangles, a new thinning operator named extended binomial is introduced, which is a general case of the binomial thinning. Compared to the binomial thinning operator, the extended binomial thinning operator has two parameters and is more flexible in modeling. Based on the proposed operator, a new integer-valued autoregressive model is introduced, which can accurately and flexibly capture the dispersed features of counting time series. Two-step conditional least squares (CLS) estimation is investigated for the innovation-free case and the conditional maximum likelihood estimation is also discussed. We have also obtained the asymptotic property of the two-step CLS estimator. Finally, three overdispersed or underdispersed real data sets are considered to illustrate a superior performance of the proposed model.

## 1. Introduction

Counting time series naturally occur in many contexts, including actuarial science, epidemiology, finance, economics, etc. The last few years have witnessed the rapid development of modeling time series of counts. One of the most common approaches for modeling integer-valued autoregressive (INAR) time series is based on thinning operators. In order to fit different kinds of situations, many corresponding operators have been developed; see [1] for a detailed discussion on thinning-based INAR models.

The most popular thinning operator is the binomial thinning operator introduced by [2]. Let *X* be a non-negative integer-valued random variable and α∈(0,1), the binomial thinning operator is defined as

(1) $$\begin{array}{c} \alpha \circ X=\sum_{i=1}^{X}B_{i},\;X>0, \end{array}$$

and 0 otherwise, where $\left\{B_{i}\right\}$ is a sequence of independent identically distributed (i.i.d.) Bernoulli random variables with fixed success probability α, and $B_{i}$ is independent of *X*. Based on the binomial thinning operator, [3,4] independently proposed an INAR(1) model as follows

(2) $$\begin{array}{c} X_{t}=\alpha \circ X_{t-1}+\epsilon_{t},\;t\in Z, \end{array}$$

where $\left\{\epsilon_{t}\right\}$ is a sequence of i.i.d. integer-valued random variables with finite mean and variance. Since this seminal work, the INAR-type models have received considerable attention. For recent literature on this topic, see [5,6], among others.

Note that $B_{i}$ in (1) follows a Bernoulli distribution, so α∘X is always less than or equal to *X*; in other words, the first part of the right side in (2) cannot be greater than $X_{t-1}$, which limits the flexibility of the model. Although it has such a shortcoming, the simple form makes it easy to estimate the parameter, and it also has many similar properties to the multiplication operator in the continuous case. For this reason, there have still been many extensions of the binomial thinning operator since its emergence. Zhu and Joe [7] proposed the expectation thinning operator, which is the generalization of binomial thinning from the perspective of a probability generating function (pgf). Although this extension is very successful, the estimation procedure is a little complicated. Compared with this extension, the thinning operator we proposed is simpler and more intuitive. For recent developments, Yang et al. [8] proposed the generalized Poisson (GP) thinning operator, which is defined by replacing $B_{i}$ with a GP counting series. Although the GP thinning operator is flexible and adaptable, we argue that it has a potential drawback: the GP distribution is not a strict probability distribution in the conventional sense. Recently, Aly and Bouzar [9] introduced a two-parameter expectation thinning operator based on a linear fractional probability generating function, which can be regarded as a general case of at least nine thinning operators. Kang et al. [10] proposed a new flexible thinning operator, which is named GSC because of three initiators of the counting series: Gómez-Déniza, Sarabia and Calderín-Ojeda.

Although the binomial thinning operator is very popular, it may not perform very well in large numerical value counting time series. This is because under such circumstances, the predicted data are often volatile, and the data are more likely to be non-stationary when the numerical value is large. We intend to establish a new thinning operator which meets the following requirements: (i) it is an extension of the binomial thinning operator; (ii) it contains two parameters to achieve flexibility, (iii) it has a simple structure and is easy to implement.

Based on the above considerations, we propose a new thinning operator based on the extended binomial (EB) distribution. The operator has two parameters: real-valued α and integer-valued *m* (0≤α≤1, m≥2), which is more flexible compared to some single parameter thinning, and the binomial thinning operator (1) can be regarded as a special case of m=2 in the EB thinning. The case of m>2 in the EB thinning usually performs better than m=2 in some large value data sets. In other words, the EB thinning alleviates the main defect of the binomial thinning to some extent. Since the EB thinning is not a special case of the expectation thinning in [9], we have further extended the framework of thinning-based INAR models to provide a new way in practical application. Therefore, an INAR(1) model is proposed based on the EB thinning operator, which is an extension of the model (2) and can more accurately and flexibly capture the dispersed features in real data.

This paper is organized as follows. In Section 2, we review the properties of the EB distribution and then introduce the EB thinning operator. Based on the new thinning operator, we propose a new INAR(1) model. In Section 3, two-step conditional least squares estimation is investigated for the innovation-free case of the model and the asymptotic property of the estimator is obtained. The conditional maximum likelihood estimation is discussed and the numerical simulations. In Section 4, we focus on forecasting and introduce two criteria to compare the prediction results for three overdispersed or underdispersed real data sets, which are considered to illustrate a better performance of the proposed model. In Section 5, we give some conclusions and related discussions.

## 2. A New INAR(1) Model

The EB distribution comes from the theory about Pascal’s triangles, which can be regarded as a multivariate case of the binomial distribution; see [11] for more details. Based on this distribution, we introduce the EB thinning operator and propose a corresponding INAR(1) model.

### 2.1. EB Distribution

The EB random variable $X_{n}\left(m,\alpha \right)$, denoted as EB(m,n,α), which is defined as follows:

(3) $$\begin{array}{c} P\left(X_{n}\left(m,\alpha \right)=r\right)=C_{m}\left(n,r\right)\alpha^{r}\beta^{(m-1)n-r},\;\;0\leq r\leq \left(m-1\right)n, \end{array}$$

where *m* and *n* are both integers satisfying m≥2 and n≥1; $C_{m}\left(n,r\right)$ can be calculated as

Cm(n,r)=∑s=0s1(−1)snsr+n−sm−1n−1,

where $s_{1}=\min\left\{n,\mathrm{integer}\;\mathrm{part}\;\mathrm{in}\;r/m\right\}$; and α and β in (3) satisfy the following restriction:

(4) $$\begin{array}{c} \beta^{m-1}+\alpha \beta^{m-2}+\alpha^{2}\beta^{m-3}+\ldots +\alpha^{m-1}=1,\;\;0\leq \alpha \leq 1,\;\;0\leq \beta \leq 1. \end{array}$$

The above restriction is equivalent to $\beta^{m}-\alpha^{m}=\beta -\alpha$. The mean and variance of EB random variables $X_{n}\left(m,\alpha \right)$ are

$$E\left(X_{n}\left(m,\alpha \right)\right)=n\alpha \frac{1-m\alpha^{m-1}}{\beta -\alpha },\;\;\mathrm{Var}\left(X_{n}\left(m,\alpha \right)\right)=n\alpha \beta \frac{1-m^{2}{\left(\alpha \beta \right)}^{m-1}}{{\left(\beta -\alpha \right)}^{2}},$$

respectively. The pgf of $X_{n}\left(m,\alpha \right)$ can be written as $G\left(t\right)=E\left(t^{X_{n}\left(m,\alpha \right)}\right)=(\frac{\beta^{m}-\alpha^{m}t^{m}}{\beta -\alpha t}{)}^{n}.$ As $X_{n}\left(m,\alpha \right)$ can be expressed as a convolution, the EB distribution has the reproductive property. Specifically, if $Y_{1},Y_{2},\ldots ,Y_{k}$ are independent random variables with $Y_{i}∼EB\left(m,n_{i},\alpha \right)$ in (3), then $\sum_{i=1}^{k}Y_{i}∼EB\left(m,\sum_{i=1}^{k}n_{i},\alpha \right)$. Notice that random variable $Y_{i}∼EB\left(2,1,\alpha \right)$ is equivalent to a Bernoulli random variable satisfying $P\left(Y_{i}=1\right)=1-P\left(Y_{i}=0\right)=1-\alpha$.

### 2.2. EB Thinning Operator

According to discussions in 2.1, we construct the EB thinning operator based on the configuration of n=1. Let $\left\{U_{i}\left(m,\alpha \right)\right\}$ be a sequence of i.i.d. random variables with common distribution EB(m,1,α), i.e., $P\left(U_{i}\left(m,\alpha \right)=z\right)=\alpha^{z}\beta^{(m-1)-z},z=0,\ldots ,m-1,$ where α and β satisfy (4). Note that the mean and variance of $U_{i}$ are

(5) $$\begin{array}{c} \mu =E\left(U_{i}\right):=\alpha \frac{1-m\alpha^{m-1}}{\beta -\alpha },\;\;\sigma^{2}=\mathrm{Var}\left(U_{i}\right):=\alpha \beta \frac{1-m^{2}{\left(\alpha \beta \right)}^{m-1}}{{\left(\beta -\alpha \right)}^{2}}. \end{array}$$

One can easily see that $\mu <\sigma^{2}$ if and only if

(6) $$\begin{array}{c} m\alpha^{m-2}\left(m\beta^{m}-\beta +\alpha \right)<1. \end{array}$$

For any 3≤m<∞, the left-hand side of (6) approaches 0 as α→0 and *m* as α→1, respectively. Hence, $\mu <\sigma^{2}$ or $\mu \geq \sigma^{2}$ is possible. When m=2, which corresponds to the binomial distribution, and (5) gives μ=α and $\sigma^{2}=\alpha \beta$ with α+β=1, then we have $\mu >\sigma^{2}$ for all 0<α<1. When α≥β and m→∞, one can easily show that μ=θ/(1−θ), $\sigma^{2}=\theta /{\left(1-\theta \right)}^{2}$, where θ=α/β. Therefore, $\mu \leq \sigma^{2}$ for all 0<θ<1 in this case.

Based on the reproductive property of the EB distribution, we define the EB thinning operator "⊚" as follows: for a non-negative integer-valued random variable *X*,

$$\left(m,\alpha \right)⊚X=\sum_{i=1}^{X}U_{i}\left(m,\alpha \right),\;X>0,$$

where (m,α)⊚X=0 if X=0. Note that the EB thinning operator reduces to the binomial operator (1) when m=2. It is easy to know that (m,α)⊚X≤X or >X, so the EB thinning operator is quite flexible when dealing with the overdispersed or underdispersed data sets.

**Remark** **1.** *The computation of (α,m) for given ($\mu ,\sigma^{2}$) is based on (4) and (5). The solution can be obtained by solving these nonlinear equations. When m=3, $\beta =(-\alpha +\sqrt{4-3\alpha^{2}})/2$ and when m=4,*

$$\begin{array}{cc} \beta \; & =\sqrt[{3}]{-\left(\frac{10\alpha^{3}}{27}-\frac{1}{2}\right)+\sqrt{{\left(\frac{10\alpha^{3}}{27}-\frac{1}{2}\right)}^{2}+{\left(\frac{2\alpha^{2}}{9}\right)}^{3}}}\\ \; & +\sqrt[{3}]{-\left(\frac{10\alpha^{3}}{27}-\frac{1}{2}\right)-\sqrt{{\left(\frac{10\alpha^{3}}{27}-\frac{1}{2}\right)}^{2}+{\left(\frac{2\alpha^{2}}{9}\right)}^{3}}}-\frac{\alpha }{3}. \end{array}$$

*For more complex cases (m≥5), we can derive the solution (α,β,m) by solving these large-scale nonlinear systems, and a more detailed calculation procedure is given in Section 3.3.*

### 2.3. EB-INAR(1) Model

Based on the EB thinning operator, we define the EB-INAR(1) model as follows:

(7) $$\begin{array}{c} X_{t}=\left(m,\alpha \right)⊚X_{t-1}+\epsilon_{t},\;t=1,2,\ldots \end{array}$$

where α∈(0,1), $\left\{X_{t}\right\}$ is a sequence of non-negative integer-valued random variables; the innovation process $\left\{\epsilon_{t}\right\}$ is a sequence of i.i.d. integer-valued random variables with finite mean and variance; and $\epsilon_{t}$ is independent of $\left\{X_{s},s<t\right\}$.

In order to obtain the estimation equations, we give some conditional or unconditional moments of the EB-INAR(1) model in the following proposition.

**Proposition** **1.** *Suppose $\left\{X_{t}\right\}$ is a stationary process defined by (7) and let μ<1; then for t≥1,*

*1.*  *$E\left(X_{t}|X_{t-1}\right)=\mu X_{t-1}+\mu_{\epsilon }$;*
*2.*  *$E\left(X_{t}\right)=\frac{\mu_{\epsilon }}{1-\mu }$;*
*3.*  *$\mathrm{Var}\left(X_{t}|X_{t-1}\right)=\sigma^{2}X_{t-1}+\sigma_{\epsilon }^{2}$;*
*4.*  *$\mathrm{Var}\left(X_{t}\right)=\frac{\sigma^{2}\mu_{\epsilon }+\sigma_{\epsilon }^{2}\left(1-\mu \right)}{{\left(1-\mu \right)}^{2}\left(1+\sigma^{2}\right)}$;*
*5.*  *$Cov\left(X_{t},X_{t-h}\right)=\mu^{h}\mathrm{Var}\left(X_{t-h}\right)$ and $Corr\left(X_{t},X_{t-h}\right)=\mu^{h},\;\mathrm{for}\;h=0,1,2,\ldots$*

*where $\mu_{\epsilon }$ and $\sigma_{\epsilon }^{2}$ are the expectation and variance of the innovation $\epsilon_{t}$, respectively.*

The proof of some of these properties mentioned above is given in Appendix A.

**Remark** **2.** *Inspired by the INAR(p) model in [12], we can further extend this model to INAR(p); the EB-INAR(p) model is defined as follows:*

$$\begin{array}{c} X_{t}=\left(m,\alpha_{1}\right)⊚X_{t-1}+\ldots +\left(m,\alpha_{p}\right)⊚X_{t-p}+\epsilon_{t},\;t=2,3,\ldots \end{array}$$

*where $\alpha_{1},\ldots ,\alpha_{p}\in \left(0,1\right)$, m is an integer satisfying m≥2, $\left\{X_{t}\right\}$ is a sequence of non-negative integer-valued random variables, the innovation process $\left\{\epsilon_{t}\right\}$ is a sequence of i.i.d. integer-valued random variables with finite mean and variance, and $\epsilon_{t}$ is independent with $\left\{X_{s},s<t\right\}$.*

We will show that the new model can accurately and flexibly capture the dispersion features of real data in Section 4.

## 3. Estimation

We use the two-step conditional least squares estimation proposed by [13] to investigate the innovation-free case and the asymptotic properties of the estimators are obtained. Conditional maximum likelihood estimation for the parametric cases are also discussed. Finally, we demonstrate the finite sample performance via simulation studies.

### 3.1. Two-Step Conditional Least Squares Estimation

Denote $\theta_{1}={\left(\mu ,\mu_{\epsilon }\right)}^{⊤}$, $\theta_{2}={\left(\sigma^{2},\sigma_{\epsilon }^{2}\right)}^{⊤}$ and $\theta ={\left(\theta_{1}^{⊤},\theta_{2}^{⊤}\right)}^{⊤}$. The two-step CLS estimation will be conducted by the following two steps.

**Step 1.1**. The estimator for $\theta_{1}.$

Let $g_{1}\left(\theta_{1},X_{t-1}\right)=E\left(X_{t}|X_{t-1}\right)=\mu X_{t-1}+\mu_{\epsilon }$, $q_{1t}\left(\theta_{1}\right)={\left(X_{t}-g_{1}\left(\theta_{1},X_{t-1}\right)\right)}^{2}.$ Let

$$Q_{1}\left(\theta_{1}\right)=\sum_{t=1}^{n}q_{1t}\left(\theta_{1}\right)$$

be the CLS criterion function. Then the CLS estimator ${\hat{\theta }}_{1,CLS}:={\left({\hat{\mu }}_{CLS},{\hat{\mu }}_{\epsilon ,CLS}\right)}^{⊤}$ of $\theta_{1}$ can be obtained by solving the score equation $\frac{\partial Q_{1}\left(\theta_{1}\right)}{\partial \theta_{1}}=0$, which implies a closed-form solution:

$${\hat{\theta }}_{1,CLS}={\left(\begin{array}{cc} \sum_{t=1}^{n}X_{t-1}^{2} & \sum_{t=1}^{n}X_{t-1}\\ \sum_{t=1}^{n}X_{t-1} & n \end{array}\right)}^{-1}\left(\begin{array}{c} \sum_{t=1}^{n}X_{t}X_{t-1}\\ \sum_{t=1}^{n}X_{t} \end{array}\right).$$

**Step 1.2**. The estimator for $\theta_{2}.$

Let $Y_{t}=X_{t}-E\left(X_{t}|X_{t-1}\right)$, $g_{2}\left(\theta_{2},X_{t-1}\right)=\mathrm{Var}\left(X_{t}|X_{t-1}\right)=$$\sigma^{2}X_{t-1}+\sigma_{\epsilon }^{2}$. Then

$$\begin{array}{cc} E(Y_{t}^{2}|X_{t-1})\; & =E(\left(X_{t}-E{\left(X_{t}|X_{t-1}\right)}^{2}\right)|X_{t-1})\\ \; & =\mathrm{Var}\left(X_{t}|X_{t-1}\right)=g_{2}\left(\theta_{2},X_{t-1}\right). \end{array}$$

Let $q_{2t}\left(\theta_{2}\right)={\left(Y_{t}^{2}-g_{2}\left(\theta_{2},X_{t-1}\right)\right)}^{2}$; then the CLS criterion function for $\theta_{2}$ can be written as

$$Q_{2}\left(\theta_{2}\right)=\sum_{t=1}^{n}q_{2t}\left(\theta_{2}\right).$$

By solving the score equation $\frac{\partial Q_{2}\left(\theta_{2}\right)}{\partial \theta_{2}}=0$, we can obtain the CLS estimator ${\hat{\theta }}_{2,CLS}:={\left({\hat{\sigma }}_{CLS}^{2},{\hat{\sigma }}_{\epsilon ,CLS}^{2}\right)}^{⊤}$ of $\theta_{2}$, which also is a closed-form solution:

$${\hat{\theta }}_{2,CLS}={\left(\begin{array}{cc} \sum_{t=1}^{n}X_{t-1}^{2} & \sum_{t=1}^{n}X_{t-1}\\ \sum_{t=1}^{n}X_{t-1} & n \end{array}\right)}^{-1}\left(\begin{array}{c} \sum_{t=1}^{n}Y_{t}^{2}X_{t-1}\\ \sum_{t=1}^{n}Y_{t}^{2} \end{array}\right).$$

**Step 2**. Estimating parameters (m,α) via the method of moments.

The estimator $\left(\hat{m},\hat{\alpha }\right)$ of (m,α), which is called a two-step CLS estimator, can be obtained by solving the following estimation equations:

(8) $$\begin{array}{c} \left\{\begin{array}{c} {\hat{\mu }}_{CLS}=\alpha \frac{1-m\alpha^{m-1}}{\beta -\alpha },\\ {\hat{\sigma }}_{CLS}^{2}=\alpha \beta \frac{1-m^{2}{\left(\alpha \beta \right)}^{m-1}}{{\left(\beta -\alpha \right)}^{2}}, \end{array}\right. \end{array}$$

where α and β satisfy (4).

Therefore, the resulting CLS estimator is ${\hat{\Theta }}_{CLS}={\left({\hat{m}}_{CLS},{\hat{\alpha }}_{CLS},{\hat{\mu }}_{\epsilon ,CLS},{\hat{\sigma }}_{\epsilon ,CLS}^{2}\right)}^{⊤}$. To study the asymptotic behaviour of the estimator, we make the following assumptions:

**Assumption** **1.** *$\left\{X_{t}\right\}$ is a stationary and ergodic process;*

**Assumption** **2.** *$EX_{t}^{4}<\infty$.*

**Proposition** **2.** *Under assumptions 1 and 2, the CLS estimator ${\hat{\theta }}_{1,CLS}$ is strongly consistent and asymptotically normal:*

$$\sqrt{n}\left({\hat{\theta }}_{1,CLS}-\theta_{1,0}\right)\overset{L}{\to }N\left(0,V_{1}^{-1}W_{1}V_{1}^{-1}\right),$$

*where $V_{1}:=E(\frac{\partial }{\partial \theta_{1}}g_{1}\left(\theta_{1,0},X_{0}\right)\frac{\partial }{\partial \theta_{1}^{⊤}}g_{1}\left(\theta_{1,0},X_{0}\right))$, $W_{1}:=E(q_{11}\left(\theta_{1,0}\right)\frac{\partial }{\partial \theta_{1}}g_{1}\left(\theta_{1,0},X_{0}\right)\frac{\partial }{\partial \theta_{1}^{⊤}}g_{1}\left(\theta_{1,0},X_{0}\right))$, and $\theta_{1,0}=\left(\mu_{0},\mu_{\epsilon_{0}}\right)$ denotes the true value of $\theta_{1}$.*

To obtain the asymptotic normality of ${\hat{\theta }}_{2,CLS}$, we make a further assumption:

**Assumption** **3.** *$EX_{t}^{6}<\infty$.*

Then we have the following proposition.

**Proposition** **3.** *Under assumptions 1 and 3, the CLS estimator ${\hat{\theta }}_{2,CLS}$ is strongly consistent and asymptotically normal:*

$$\sqrt{n}\left({\hat{\theta }}_{2,CLS}-\theta_{2,0}\right)\overset{L}{\to }N\left(0,V_{2}^{-1}W_{2}V_{2}^{-1}\right),$$

*where $V_{2}:=E(\frac{\partial }{\partial \theta_{2}}g_{2}\left(\theta_{2,0},X_{0}\right)\frac{\partial }{\partial \theta_{2}^{⊤}}g_{2}\left(\theta_{2,0},X_{0}\right))$, $W_{2}:=E(q_{21}\left(\theta_{2,0}\right)\frac{\partial }{\partial \theta_{2}}g_{2}\left(\theta_{2,0},X_{0}\right)\frac{\partial }{\partial \theta_{2}^{⊤}}g_{2}\left(\theta_{2,0},X_{0}\right))$, and $\theta_{2,0}=\left(\sigma_{0}^{2},\sigma_{\epsilon_{0}}^{2}\right)$ denotes the true value of $\theta_{2}$.*

Based on Propositions 2 and 3 and Theorem 3.2 in [14], we have the following proposition.

**Proposition** **4.** *Under assumptions 1 and 3, the CLS estimator ${\hat{\theta }}_{CLS}={\left({\hat{\theta }}_{1,CLS},{\hat{\theta }}_{2,CLS}\right)}^{⊤}$ is strongly consistent and asymptotically normal:*

$$\begin{array}{c} \sqrt{n}\left({\hat{\theta }}_{CLS}-\theta_{0}\right)\overset{L}{\to }N\left(0,\Omega \right), \end{array}$$

*where*

$$\begin{array}{c} \Omega =\left(\begin{array}{cc} V_{1}^{-1}W_{1}V_{1}^{-1} & V_{1}^{-1}MV_{2}^{-1}\\ V_{2}^{-1}M^{⊤}V_{1}^{-1} & V_{2}^{-1}W_{2}V_{2}^{-1} \end{array}\right), \end{array}$$

*$M=E(\sqrt{q_{11}\left(\theta_{1,0}\right)q_{21}\left(\theta_{2,0}\right)}\frac{\partial }{\partial \theta_{1}}g_{1}\left(\theta_{1,0},X_{0}\right)\frac{\partial }{\partial \theta_{2}^{⊤}}g_{2}\left(\theta_{2,0},X_{0}\right))$, and $\theta_{0}={\left(\theta_{1,0},\theta_{2,0}\right)}^{⊤}$ denotes the true value of θ.*

We do the following preparation to establish Proposition 5. Based on (5), solve the equation about (m,α), and denote the solution as $\left(h_{1}\left(\mu ,\sigma^{2}\right),h_{2}\left(\mu ,\sigma^{2}\right)\right)$. Let

(9) $$\begin{array}{c} D=D\left(\mu ,\sigma^{2}\right)=\left(\begin{array}{cc} \partial h_{1}/\partial \mu & \partial h_{1}/\partial \sigma^{2}\\ \partial h_{2}/\partial \mu & \partial h_{2}/\partial \sigma^{2} \end{array}\right). \end{array}$$

Based on Proposition 4, we state the strong consistency and asymptotic normality of ${\left(\hat{m},\hat{\alpha }\right)}^{⊤}$ in the following proposition.

**Proposition** **5.** *Under assumptions 1 and 3, the CLS estimator ${\left({\hat{m}}_{CLS},{\hat{\alpha }}_{CLS}\right)}^{⊤}$ is strongly consistent and asymptotically normal:*

$$\begin{array}{c} \sqrt{n}\left(\begin{array}{c} {\hat{m}}_{CLS}-m_{0}\\ {\hat{\alpha }}_{CLS}-\alpha_{0} \end{array}\right)\overset{L}{\to }N\left(0,D\Sigma D^{⊤}\right), \end{array}$$

*where D is given in (9); $\Sigma =diag(IV_{1}^{-1}W_{1}V_{1}^{-1}I^{⊤},IV_{2}^{-1}W_{2}V_{2}^{-1}I^{⊤})$ with I=(1,0); $m_{0}$ and $\alpha_{0}$ denote the true values of m and α, respectively.*

The brief proofs of Propositions 2–5 are given in Appendix A.

### 3.2. Conditional Maximum Likelihood Estimation

We maximize the likelihood function with respect to the model parameters θ=(m,α,δ) to get the conditional maximum likelihood (CML) estimate of the parametric case

$$\begin{array}{cc} L(X_{1}=x_{1},\ldots ,X_{N}=x_{N}|\theta )\; & =P_{\theta }\left(X_{1}=x_{1}\right)\prod_{i=1}^{N}P_{\theta }\left(X_{i}=x_{i}|X_{i-1}=x_{i-1},\ldots ,X_{1}=x_{1}\right)\\ \; & =P_{\theta }\left(X_{1}=x_{1}\right)\prod_{i=1}^{N}P_{\theta }\left(X_{i}=x_{i}|X_{i-1}=x_{i-1}\right), \end{array}$$

where δ is the parameter of $\epsilon_{i}$, $P_{X_{1}}$ is the pmf for $X_{1}$ and $P_{\theta }\left(X_{i+1}|X_{i}\right)$ is the conditional pmf. Since the marginal distribution is difficult to obtain in general, a simple approach is conditional on the observed $X_{1}$. By essentially ignoring the dependency on the initial value and considering the CML estimate given $X_{1}$ as an estimate for θ by maximizing the conditional log-likelihood

$$\begin{array}{c} l\left(X_{1}=x_{1},\ldots ,X_{N}=x_{N}|\theta \right)=\sum_{i=2}^{N}\log P_{\theta }\left(X_{i}|X_{i-1}\right) \end{array}$$

over Θ, we denote the CML estimate by $\hat{\theta }=\left(\hat{m},\hat{\alpha },\hat{\delta }\right)$. The log-likelihood function is as follows:

$$\begin{array}{cc} l(X_{1}=x_{1},\ldots ,X_{N}=x_{N}|\theta )\; & =\sum_{i=2}^{N}\log\{\sum_{w=0}^{\min\{\left(m-1\right)x_{i-1},x_{i}\}}C_{m}\left(x_{i-1},w\right)\alpha^{w}\beta^{\left(m-1\right)x_{i-1}-w}\cdot P\left(\epsilon_{i}=x_{i}-w\right)\}, \end{array}$$

where α and β satisfy (4); $\epsilon_{i}$ follows a non-negative discrete distribution with a parameter δ. In what follows, we consider two cases: m=3,4.

**Case 1**: For *m* = 3 with Poisson innovation, i.e., $\epsilon_{t}∼P\left(\delta \right)$.

l(X1=x1,…,XN=xN|θ)=∑i=2Nlog{∑w=0min{2xi−1,xi}∑t1=max{0,xi−1−w}xi−1−w2xi−1t1xi−1−t12xi−1−2t1−w·(β2)t1(αβ)2xi−1−2t1−w(α2)t1−xi−1+wδ(xi−w)(xi−w)!e−δ},

where β is given in Remark 1.

**Case 2**: For *m* = 4 with geometric innovation, i.e., $\epsilon_{t}∼Ge\left(\delta \right)={\left(1-\delta \right)}^{k}\delta \;\mathrm{for}\;k=0,1,2,\ldots$

l(X1=x1,…,XN=xN|θ)=∑i=2Nlog{∑w=0min{3xi−1,xi}∑t1=xi−1−wxi−1−w3∑t2=max{0,2xi−1−2t1−w}3xi−1−3t1−w2xi−1t1xi−1−t1t2·xi−1−t1−t23xi−1−3t1−2t2−w(β3)t1(αβ2)t2(α2β)3xi−1−3t1−2t2−w·(α3)2t1+t2−2xi−1+w(1−δ)(xi−w)δ},

where β is given in Remark 1. For higher order *m*, the formula is a little tedious, which is omitted here. For the estimate of EB-INAR(*p*), the CML estimation is too complicated, but the two-step CLS estimation is quite feasible, the procedure is similar to the case of p=1. For this reason, we only consider the case of EB-INAR(1) in simulation studies.

### 3.3. Simulation

A Monte Carlo simulation study was conducted to evaluate the finite sample performance of the estimator. For CLS estimation, we used the package BB in R for solving and optimizing large-scale nonlinear systems to solve Equations (4) and (8). For CML estimation, we used the package maxLik in R to maximize the log-likelihood function.

We considered the following configurations of the parameters:

- Poisson INAR(1) models with $\theta ={\left(m,\alpha ,\delta \right)}^{⊤}:$
  $\left(A1\right)={\left(3,0.2,1\right)}^{⊤};\;\;\;\left(A2\right)={\left(3,0.1,0.5\right)}^{⊤};\;\;\;\left(A3\right)={\left(4,0.2,1\right)}^{⊤};\;\;\;\left(A4\right)={\left(4,0.1,0.5\right)}^{⊤};$
- Geometric INAR(1) models with $\theta ={\left(m,\alpha ,\delta \right)}^{⊤}:$
  $\left(B1\right)={\left(3,0.3,0.5\right)}^{⊤};\;\;\;\left(B2\right)={\left(3,0.4,2/3\right)}^{⊤};\;\;\;\left(B3\right)={\left(4,0.3,0.5\right)}^{⊤};\;\;\;\left(B4\right)={\left(4,0.4,2/3\right)}^{⊤}.$

In simulations, we chose sample sizes *n* = 100, 200 and 400 with M=500 replications for each choice of parameters. The root mean squared error (RMSE) was calculated to evaluate the performance of the estimator according to the following formula: $\mathrm{RMSE}=\sqrt{\frac{1}{M-1}\sum_{j=1}^{M}{\left({\hat{\xi }}_{j}-\xi_{0}\right)}^{2}},$ where ${\hat{\xi }}_{j}$ is the estimator of $\xi_{0}$ in the *j*th replication.

For the CLS estimate, the solutions of (4) and (8) are sensitive to $\hat{\mu }$ and ${\hat{\sigma }}^{2}$, so we adopted the following estimation procedure. First, calculate 500 groups of $\hat{\mu }$ and ${\hat{\sigma }}^{2}$ estimates, then use the mean values of $\hat{\mu }$ and ${\hat{\sigma }}^{2}$ to solve the Equations (4) and (8). The simulation results of CLS are summarized in Table 1. We found that the estimation values are closer to the true value and the values of RMSE gradually decrease as the sample size increases.

As it is a little difficult to estimate the parameter *m* in CML estimation, we considered *m* as known. The simulation results of CML estimators are given in Table 2. For all cases, all estimates generally show small values of RMSE, and the values of RMSE gradually decrease as the sample size increases.

## 4. Real Data Examples

In this section, three real data sets, including overdispersed and underdispersed settings, are considered to illustrate the better performance of the proposed model. The first example is overdispersed crime data in Pittsburgh; the second is overdispersed stock data in New York Stock Exchange (NYSE); and the third is underdispersed crime data in Pittsburgh, which was also analyzed by [15]. As is well known, in time series analysis, forecasting is very important in model evaluation. We first introduce two criteria on forecasting, and other preparations.

### 4.1. Forecasting

Before introducing the evaluation criterion, we briefly introduce the basic procedure as follows: First, we divide the $n_{1}+n_{2}$ data into two parts, the training set with the first $n_{1}$ data and the prediction set with the last $n_{2}$ data. The training set is used to estimate the parameters and evaluate the fitness of the model. Then we can evaluate the efficiency of each model by comparing the following criteria between prediction data and the real data in the prediction set.

Similar to the procedure in [16], which performs an out-of-sample experiment to compare forecasting performances of two model-based bootstrap approaches, we introduce the forecasting procedure as follows: For each $t=\left(n_{1}+1\right),\ldots ,\left(n_{1}+n_{2}-5\right)$ we estimate an INAR(1) model for the data $x_{1},\ldots ,x_{t}$, then we use the fitted result based on $x_{1},\ldots ,x_{t}$ to generate the next five forecasts, which is called the 5-step ahead forecast $x_{t+1}^{F},\ldots ,x_{t+5}^{F}$ for each *t* in $\left\{\left(n_{1}+1\right),\ldots ,\left(n_{1}+n_{2}-5\right)\right\}$, where $x_{t}^{F}$ is the forecast at time *t*. In this way we obtain many sequences of 1,2,…,5 step-ahead forecasts, finally we replicate the whole procedure *P* times. Then we can evaluate the point forecast accuracy by the forecast mean square error (FMSE) defined as

$$\mathrm{FMSE}=\frac{1}{P}\sum_{i=(n_{1}+1)}^{n_{2}}{\left(x_{i}-{\bar{x}}_{i}^{F}\right)}^{2},$$

and forecast mean absolute error (FMAE) defined as

$$\mathrm{FMAE}=\frac{1}{P}\sum_{i=(n_{1}+1)}^{n_{2}}\left|x_{i}-{\bar{x}}_{i}^{F}\right|,$$

where $x_{i}$ is the true value of the data, ${\bar{x}}_{i}^{F}$ is the mean of all the forecasts at *i* and *P* is the number of replicates.

### 4.2. Overdispersed Cases

We consider two overdispersed data sets, the first one contains 144 observations and represents monthly tallies of crime data from the Forecasting Principles website http://www.forecastingprinciples.com, and these crimes are reported in the police car beats in Pittsburgh from January 1990 to December 2001; the second one is Empire District Electric Company (EDE) data set from the Trades and Quotes (TAQ) set in NYSE, which contains 300 observations, and it was also analyzed by [17].

#### 4.2.1. P1V Data

**Figure 1:**
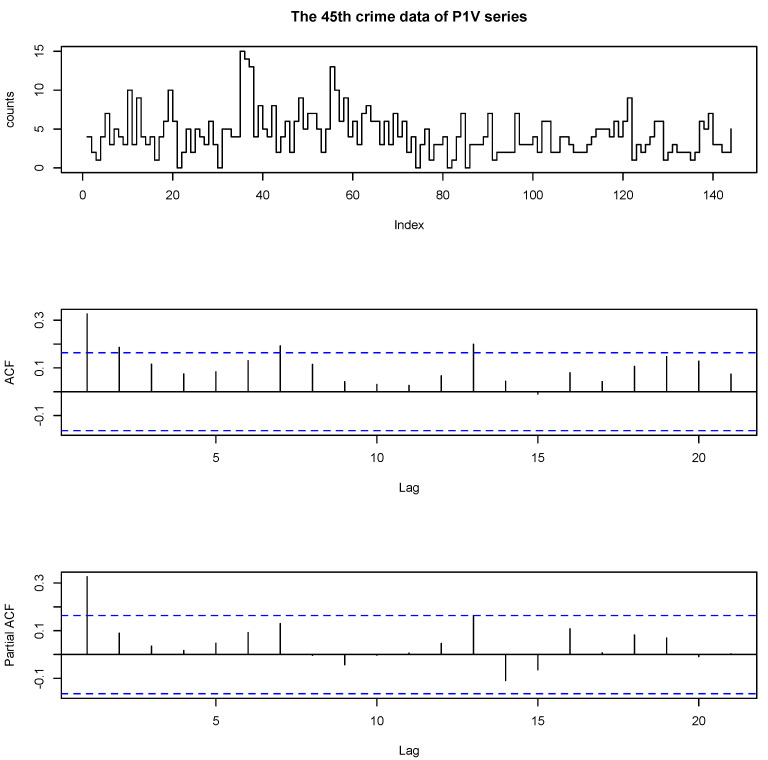
The data, autocorrelation function (ACF) and partial autocorrelation function (PACF) of 45th P1V series.

The 45th P1V (Part 1 Violent Crimes) data set contains crimes of murder, rape, robbery and other kinds; see more details in the data dictionary on the Forecasting Principles website. Figure 1 plots the time series plot, the autocorrelation function (ACF) and the partial autocorrelation function (PACF) of 45th data of P1V series, respectively. The maximum value of the data is 15 and the minimum is 0; the mean is 4.3333; the variance is 7.4685. From the ACF plot, we found that the data are dependent. From the PACF plots, we can see that only the first sample is significant, which strongly suggests an INAR(1) model.

First, we divided the data set into two parts–the training set with the first $n_{1}=134$ counting data and the prediction set with the last $n_{2}=10$ data. We fit the training set by the following models: expectation thinning INAR(1) (ETINAR(1)) model in [9], GSC thinning INAR(1) (GSCINAR(1)) model in [10], the binomial thinning INAR(1) model and EB thinning EB-INAR(1) models with m=3,4. According to the mean and variance of P1V data, we used one of the most common settings–geometric distribution–as the distribution of the innovation in above models.

In order to compare the effectiveness of the models, we consider the following evaluation criteria: (1) AIC. (2) The mean and standard error of Pearson residual $r_{t}$ and its related Ljung–Box statistics, where the Pearson residuals are defined as

$$r_{t}=\frac{X_{t}-\hat{\mu }X_{t-1}-{\hat{\mu }}_{\epsilon }}{{\left[{\hat{\sigma }}^{2}X_{t-1}+{\hat{\sigma }}_{\epsilon }^{2}\right]}^{\frac{1}{2}}},\;\;t=1,2,\ldots ,$$

where $\hat{\mu }$ and ${\hat{\sigma }}^{2}$ are the estimated expectation and variance for related thinning operators, respectively. (3) Three goodness-of-fit statistics: RMS (root mean square error), MAE (mean absolute error) and MdAE (median absolute error), where the error is defined by $X_{t}-E\left(X_{t}|X_{t-1}\right)$, $t=1,\ldots ,n_{1}$. (4) The mean of the data $\hat{\bar{x}}$ on the training set calculated by the estimated results.

Next, focusing on forecasting, we generated P=100 replicates based on the training set for each model. Then we calculated the FMSE and FMAE for each model.

All results of the fitted models are given in Table 3. There is no evidence of any correlation within the residuals of all five models, which is also supported by the Ljung–Box statistic based on 15 lags (because $\chi_{0.05}^{2}\left(14\right)=23.6847$). There were no significant differences for the RMS, MAE, MdAE and $\hat{\bar{x}}$ values (the true mean of the 134 training set was 4.3880) of the models. In other words, no model performed the best in terms of these four criteria, so we also considered AIC. Since the CML estimator cannot be adopted in GSCINAR(1), one can only compare other criteria.

Considering the fitness on the training set, the EB-INAR(1) with m=3 has the smallest AIC, EB-INAR(1) with m=4 has almost the same AIC as m=3. For the results on forecasting, EB-INAR(1) with m=4 has the smallest FMSE and the second smallest FMAE among all models. EB-INAR(1) with m=3 has the second smallest FMSE and the smallest FMAE. Based on these results, we conclude that EB-INAR(1) with m=3,4 performs better than INAR(1), ETINAR(1) and GSCINAR(1).

#### 4.2.2. Stock Data

We analyzed another overdispersed data set of Empire District Electric Company (EDE) from the Trades and Quotes (TAQ) data set in NYSE. The data are about the number of trades in 5 min intervals between 9:45 a.m. and 4:00 p.m. in the first quarter of 2005 (3 January–31 March 2005, 61 trading days). Here we analyze a portion of the data between first to fourth trading days. As there are 75 5 min intervals per day, the sample size was *T* = 300.

**Figure 2:**
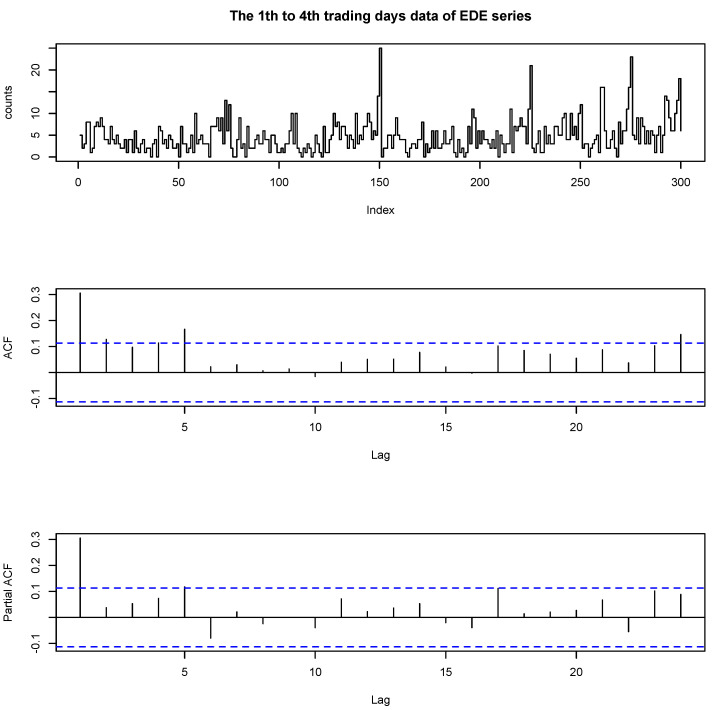
The data, ACF and PACF of first to fourth trading days of EDE series.

Figure 2 plots the time series plot, the ACF and the PACF of the EDE series. The maximum value of the data is 25 and the minimum is 0; the mean is 4.6933; and the variance is 14.1665. It seems that the series is not completely stationary with several outliers or influential observations based on the time series plot. Zhu et al. [18] analyzed the Poisson autoregression for the stock transaction data with extreme values, which can be considered in the current setting. From the ACF plot, we found that the data are dependent. From the PACF plots, we can see that only the first sample is significant, which strongly suggests an INAR(1) model. We used the same procedures and criteria as before. We used the geometric distribution as the distribution of the innovation in above models.

First divide the data set into two parts–the training set with the first $n_{1}=270$ data and the prediction set with the last $n_{2}=30$ data. All results of the fitted models are given in Table 4. Among all models, EB-INAR(1) with m=4 has the smallest AIC, and there is no evidence of any correlation within the residuals of all five models, which is also supported by the Ljung–Box statistic based on 15 lags. There are no significant differences for the RMS, MAE, MdAE and $\hat{\bar{x}}$ values (the true mean of the 270 training set was 4.3407) of all considered models. For the results of prediction, EB-INAR(1) with m=4 has the smallest FMSE and FMAE among all models. Based on the above results, we conclude that EB-INAR(1) with m=4 performs best for this data set.

### 4.3. Underdispersed Case

**Figure 3:**
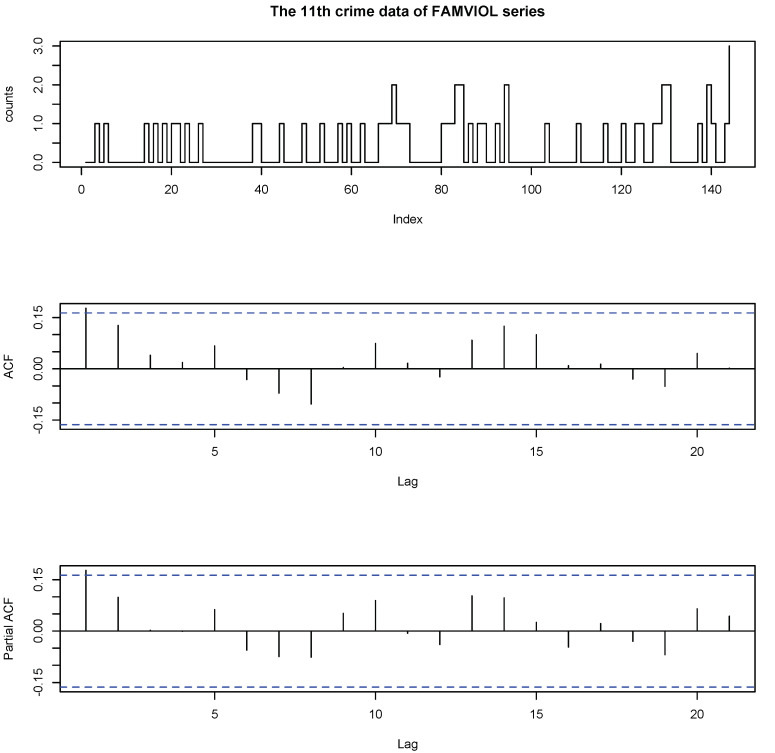
The data, ACF and PACF of the 11th data set of the FAMVIOL series.

The 11th FAMVIOL data set contains the crimes of family violence, which can also be obtained from the Forecasting Principles website. Figure 3 plots the time series plot, the ACF and the PACF of the 11th data set of FAMVIOL series. The maximum value of the data is 3 and the minimum is 0; the mean is 0.4027; and the variance is 0.3820. We use the procedures and criteria in Section 4.2.1 to compare different models. According to the mean and the variance of FAMVIOL data, we use one of the most common settings-Poisson distribution as the distribution of the innovation in above models.

All results of the fitted models are given in Table 5. There is no evidence of any correlation within the residuals of all five models, which is also supported by the Ljung–Box statistic based on 15 lags. There are no significant differences about the criteria on the fitness and forecasting of all models. ETINAR(1) with the biggest AIC, performed the worst in these models.

Now let us have a brief summary. For the P1V data and stock data, which are overdispersed with slightly high-count data, the EB-INAR(1) of m>2 is obviously better than m=2. For the FAMVIOL data, which is underdispersed with small-count data, the EB-INAR(1) with m>2 is also competitive.

## 5. Conclusions

This paper proposes an EB-INAR(1) model based on the newly constructed EB thinning operator, which is an extension of the thinning-based INAR models. We gave the estimation method for parameters and established the asymptotic properties of the estimators for the innovation-free case. Based on the simulations and real data analysis, the EB-INAR(1) model can accurately and flexibly capture the dispersion features of the data, which shows its effectiveness and practicality. Compared with other models, such as ETINAR(1) and GSCINAR(1), our model is competitive.

We point out that many existing integer-valued models can be generalized by replacing the binomial thinning operator with the EB thinning operator, such as those models in [19,20,21,22,23]. In addition, we can extend the considered first-order INAR model to the higher-order one. More research will be studied in the future.

## Figures and Tables

**Table 1:** Means of estimates, RMSEs (within parentheses) by CLS.

Case	*n*	$\hat{m}$	$\hat{\alpha }$	$\hat{\delta }$
A1	100	2.9100	0.1758	1.0220(0.1595)
	200	2.9219	0.1931	1.0142(0.1148)
	400	2.9995	0.1984	1.0090(0.0771)
A2	100	2.7628	0.0886	0.5014(0.0893)
	200	2.8169	0.0873	0.5049(0.0668)
	400	2.9132	0.0942	0.5004(0.0449)
A3	100	3.7885	0.1713	1.0265(0.1594)
	200	3.8450	0.1902	1.0165(0.1127)
	400	3.8957	0.1952	1.0113(0.0789)
A4	100	3.8421	0.0912	0.5059(0.0912)
	200	3.8483	0.0912	0.5031(0.0603)
	400	3.9590	0.0981	0.5012(0.0439)
B1	100	2.9074	0.3122	0.4981(0.0511)
	200	2.9588	0.3115	0.5008(0.0365)
	400	2.9858	0.3087	0.4984(0.0270)
B2	100	3.0719	0.3841	0.6578(0.0553)
	200	3.0523	0.3877	0.6569(0.0400)
	400	3.0986	0.3924	0.6600(0.0307)
B3	100	3.6127	0.2703	0.4962(0.0536)
	200	3.7937	0.2961	0.4980(0.0404)
	400	3.9217	0.2984	0.4970(0.0269)
B4	100	3.9575	0.3935	0.6417(0.0653)
	200	4.0388	0.3929	0.6556(0.0478)
	400	4.0027	0.3953	0.6606(0.0353)

Note: RMSE, root mean squared error.

**Table 2:** Means of estimates, RMSEs (within parentheses) by CML.

Case	*n*	100	200	400
A1	$\hat{\alpha }$	0.1858(0.0656)	0.1927(0.0476)	0.1970(0.0347)
	$\hat{\delta }$	1.0070(0.1420)	1.0043(0.1057)	1.0057(0.0806)
A2	$\hat{\alpha }$	0.1041(0.0575)	0.1027(0.0453)	0.0973(0.0357)
	$\hat{\delta }$	0.4939(0.0804)	0.4964(0.0593)	0.4973(0.0422)
A3	$\hat{\alpha }$	0.1827(0.0592)	0.1923(0.0406)	0.1966(0.0306)
	$\hat{\delta }$	1.0186(0.1358)	1.0030(0.1059)	1.0002(0.0789)
A4	$\hat{\alpha }$	0.0938(0.0500)	0.0940(0.0414)	0.0987(0.0342)
	$\hat{\delta }$	0.4993(0.0790)	0.5052(0.0583)	0.4967(0.0420)
B1	$\hat{\alpha }$	0.2982(0.0456)	0.2980(0.0329)	0.2992(0.0237)
	$\hat{\delta }$	0.5086(0.0469)	0.5013(0.0323)	0.5016(0.0224)
B2	$\hat{\alpha }$	0.3888(0.0455)	0.3949(0.0331)	0.3980(0.0222)
	$\hat{\delta }$	0.6685(0.0501)	0.6681(0.0374)	0.6664(0.0256)
B3	$\hat{\alpha }$	0.2904(0.0441)	0.2958(0.0276)	0.2990(0.0219)
	$\hat{\delta }$	0.5039(0.0480)	0.5003(0.0338)	0.5006(0.0264)
B4	$\hat{\alpha }$	0.3867(0.0348)	0.3940(0.0240)	0.3965(0.0163)
	$\hat{\delta }$	0.6650(0.0539)	0.6674(0.0382)	0.6662(0.0271)

Note: RMSE, root mean squared error.

**Table 3:** Fitting results, AIC and some characteristics of P1V data.

Model	Estimates	AIC	$\bar{r_{t}}$	std($r_{t}$)	Ljung-Box	RMS	MAE	MdAE	$\hat{\bar{x}}$	FMSE	FMAE
INAR(1)	$\hat{\alpha }=0.3955$	641.9136	0.0020	0.8008	12.5545	2.6384	2.0236	1.5828	4.3811	0.3988	0.1809
	$\hat{\delta }=0.2741$										
	$\hat{q}=0.4942$										
ETINAR(1)	$\hat{r}=0.3739$	639.3243	0.0134	0.8392	16.6092	2.6736	2.0628	1.6958	4.3758	0.3847	0.1821
	$\hat{\delta }=0.3112$										
GSCINAR(1)	$\hat{\gamma }=0.8944$	--------	0.0005	0.6805	12.7469	2.6304	2.0027	1.7395	4.3838	0.4115	0.1935
	$\hat{\delta }=0.2515$										
EB-INAR(1) with m=3	$\hat{\alpha }=0.3299$	636.2386	0.0097	0.8525	15.7722	2.6666	2.0563	1.7167	4.3771	0.3770	0.1748
	$\hat{\delta }=0.3050$										
EB-INAR(1) with m=4	$\hat{\alpha }=0.3164$	636.2887	0.0145	0.8526	17.0785	2.6789	2.0673	1.6815	4.3756	0.3589	0.1791
	$\hat{\delta }=0.3156$										

**Table 4:** Fitting results, AIC and some characteristics of EDE data.

Model	Estimates	AIC	$\bar{r_{t}}$	std($r_{t}$)	Ljung-Box	RMS	MAE	MdAE	$\hat{\bar{x}}$	FMSE	FMAE
INAR(1)	$\hat{\alpha }=0.2457$	1341.648	0.0003	0.8590	13.0696	3.3407	2.4817	2.0075	4.3358	10.2602	1.2368
	$\hat{\delta }=0.2341$										
	$\hat{q}=0.3204$										
ETINAR(1)	$\hat{r}=0.4125$	1339.397	0.0041	0.8811	16.4070	3.3570	2.4910	2.0534	4.3357	10.6103	1.2688
	$\hat{\delta }=0.2533$										
GSCINAR(1)	$\hat{\gamma }=0.9578$	--------	−0.0003	0.8027	13.1596	3.3397	2.4812	2.0409	4.3361	10.4577	1.2362
	$\hat{\delta }=0.2284$										
EB-INAR(1) with m=3	$\hat{\alpha }=0.2200$	1338.071	0.0018	0.8801	14.5878	3.3477	2.4863	2.0299	4.3352	10.1038	1.2317
	$\hat{\delta }=0.2448$										
EB-INAR(1) with m=4	$\hat{\alpha }=0.2194$	1337.554	0.0027	0.8838	15.3238	3.3514	2.4880	2.0224	4.3350	10.0463	1.2279
	$\hat{\delta }=0.2486$										

**Table 5:** Fitting results, AIC and some characteristics of FAMVIOL data.

Model	Estimates	AIC	$\bar{r_{t}}$	std($r_{t}$)	Ljung-Box	RMS	MAE	MdAE	$\hat{\bar{x}}$	FMSE	FMAE
INAR(1)	$\hat{\alpha }=0.1750$	206.5983	−0.0005	0.9157	8.6672	0.5596	0.4940	0.4851	0.3759	0.1111	0.0790
	$\hat{\delta }=0.3101$										
	$\hat{q}=0.1774$										
ETINAR(1)	$\hat{r}=0.0267$	208.5826	−0.0003	0.9122	8.6344	0.5596	0.4939	0.4866	0.3759	0.1205	0.0837
	$\hat{\delta }=0.3092$										
GSCINAR(1)	$\hat{\gamma }=0.9741$	--------	0.0023	0.8609	8.3735	0.5595	0.4932	0.4944	0.3759	0.1020	0.0814
	$\hat{\delta }=0.3045$										
EB-INAR(1) with m=3	$\hat{\alpha }=0.1505$	206.5335	0.0007	0.9005	8.5485	0.5595	0.4935	0.4900	0.3757	0.1135	0.0792
	$\hat{\delta }=0.3068$										
EB-INAR(1) with m=4	$\hat{\alpha }=0.1371$	206.8414	−0.0014	0.8943	8.4928	0.5597	0.4944	0.4802	0.3759	0.1149	0.0808
	$\hat{\delta }=0.3131$										

## Appendix A

**Proof of Proposition 1.** Since 1–4 are easy to verify, we only prove 5. By the law of total covariance, we have

$$\begin{array}{cc} Cov(X_{t},X_{t-h})\; & =Cov(E\left(X_{t}|X_{t-1},\ldots \right),E\left(X_{t-h}|X_{t-1},\ldots \right))+E(Cov\left(X_{t},X_{t-h}|X_{t-1},\ldots \right))\\ \; & =Cov(\mu X_{t-1}+\mu_{\epsilon },X_{t-h})\\ \; & +E(E\left(X_{t}-E\left(X_{t}|X_{t-1},\ldots \right)\right)\left(X_{t-h}-E\left(X_{t-h}|X_{t-1},\ldots \right)\right)|X_{t-1},\ldots )\\ \; & =\mu \cdot Cov(X_{t-1},X_{t-h})+0\\ \; & =\cdot \cdot \cdot \\ \; & =\mu^{h}\cdot \mathrm{Var}\left(X_{t-h}\right). \end{array}$$

Thus, the autocorrelation function $Corr\left(X_{t},X_{t-h}\right)=\mu^{h}$. □

**Proof of Propositions 2 and 3.** Propositions 2 and 3 are similar to Theorems 1 and 2 in [8], which can be proved by verifying the regularity conditions of Theorems 3.1 and 3.2 in [24]. For instance, in the proof of Proposition 2, the partial derivatives $\partial g\left(\alpha ,F_{m-1}\right)/\partial \alpha_{i}$ have finite fourth moments in [24], which correspond to Assumption 2 in Section 3.1, $u_{m}^{2}\left(\alpha \right)$ in [24] is corresponds to $q_{1t}\left(\theta_{1}\right)$ in Step 1.1. Hence, Proposition 2 can be regarded as a direct conclusion of Theorem 3.2. Besides, the proof of Proposition 3 is similar to Proposition 2; the procedure is almost the same as Theorem 3.2 in [24]. □

**Proof of Proposition 4.** Similarly to the Theorem in [25], based on Theorem 3.2 in [14], we have

$$\begin{array}{c} \sqrt{n}\left({\hat{\theta }}_{CLS}-\theta_{0}\right)\overset{L}{\to }N\left(0,\Omega \right), \end{array}$$

where

$$\begin{array}{c} \Omega =\left(\begin{array}{cc} V_{1}^{-1}W_{1}V_{1}^{-1} & V_{1}^{-1}MV_{2}^{-1}\\ V_{2}^{-1}M^{⊤}V_{1}^{-1} & V_{2}^{-1}W_{2}V_{2}^{-1} \end{array}\right). \end{array}$$

Based on the proof of the Theorem in [25], $\sqrt{q_{1t}\left(\theta_{1}\right)}$ corresponds to $u_{t}$ and $\sqrt{q_{2t}\left(\theta_{2}\right)}$ corresponds to $U_{t}$ in [25]. Based on the result of $V_{1},W_{1}$ in Proposition 2 and $V_{2},W_{2}$ in Proposition 3, we can obtain $M=E(\sqrt{q_{11}\left(\theta_{1,0}\right)q_{21}\left(\theta_{2,0}\right)}\frac{\partial }{\partial \theta_{1}}g_{1}\left(\theta_{1,0},X_{0}\right)\frac{\partial }{\partial \theta_{2}^{⊤}}g_{2}\left(\theta_{2,0},X_{0}\right))$. □

**Proof of Proposition 5.** Since the solutions ($h_{1}\left(\mu ,\sigma^{2}\right)$, $h_{2}\left(\mu ,\sigma^{2}\right)$) about (m,α) in (3.1) are real-valued and have a nonzero differential, Proposition 5 is an application of the δ-method, for example, which can be found in Theorem A on p.122 of [26]. □
